# Evaluation of indocyanine green fluorescence guidance in minimally invasive partial nephrectomy stratified by tumor anatomical complexity: a single-center retrospective study

**DOI:** 10.3389/fsurg.2026.1887270

**Published:** 2026-09-18

**Authors:** Zuodong Yang, Siwei Chen, Jia Luo, Xin Xie, Haichao Yuan, Hang Li, Liangkuan Bi, Xing Fu, Guanqun Wang

**Affiliations:** 1 Department of Urology, Peking University Shenzhen Hospital, Shenzhen, China; 2 Shenzhen Key Laboratory of Male Reproductive Medicine and Genetics, Institute of Urology, Peking University Shenzhen Hospital, Shenzhen, China; 3 Shenzhen Clinical Research Center for Urology and Nephrology, Peking University Shenzhen Hospital, Shenzhen, China

**Keywords:** fluorescence-guided surgery, indocyanine green, nephrometry score, partial nephrectomy, renal cell carcinoma, robot-assisted surgery

## Abstract

**Objectives:**

To evaluate the impact of indocyanine green (ICG) fluorescence guidance on surgical efficiency and renal function during partial nephrectomy (PN), across different tumor anatomical complexities.

**Methods:**

We retrospectively reviewed 77 consecutive patients with renal cell carcinoma who underwent minimally invasive PN (MIPN, predominantly robot-assisted, *n* = 76) between April 2024 and August 2025. Patients were grouped into ICG (*n* = 31) and non-ICG (*n* = 46) cohorts. Outcomes were compared for the whole cohort and stratified by RENAL score (low-complexity < 9 vs. moderate-to-high complexity ≥ 9). Multivariable linear regression and Pearson correlation analyses were performed.

**Results:**

Baseline characteristics were comparable. Overall, operative time was significantly shorter in the ICG group in unadjusted (139.84 vs. 163.57 min; *P* = 0.017) and multivariable regression analyses adjusted for tumor size, RENAL score, and surgical approach (*B* = −23.808, 95% CI: −41.685 to −5.931, *P* = 0.010). In the low-complexity subgroup (RENAL score < 9), ICG significantly reduced operative time (*P* = 0.038), but this association attenuated after adjusting for tumor size (*P* = 0.099). In moderate-to-high complexity cases, no significant difference was detected in unadjusted (*P* = 0.357) or adjusted (*B* = −28.960, *P* = 0.067, 95% CI: −60.086 to 2.166) analyses, while larger tumor size independently predicted longer operative time (*B* = 12.100, *P* = 0.014, 95% CI: 2.306 to 21.894). Furthermore, in this high-complexity cohort, shorter operative time was significantly correlated with higher preservation of estimated glomerular filtration rate (eGFR) on postoperative day 1 (*B* = −0.002, *P* = 0.029, 95% CI: −0.003 to −1.920 × 10⁻⁴). Warm ischemia time, blood loss, and complications were similar between groups (all *P* > 0.05).

**Conclusion:**

The primary value of ICG fluorescence guidance in MIPN lies in optimizing intraoperative surgical efficiency and shortening operative time; exploratory stratified analyses showed significant operative time reduction with ICG in low-complexity tumors, whereas no significant difference occurred in high-complexity cases. The operative efficiency benefit of ICG in complex cases remains unconfirmed due to limited statistical power. Whether ICG guidance effectively preserves or improves early postoperative renal function remains unknown and requires further investigation.

## 1 Introduction

Partial nephrectomy (PN) is the gold-standard surgical approach for early and selected locally advanced renal cell carcinoma (RCC) (1–3), which provides superior renal function preservation (4, 5) and favorable long-term oncological outcomes (6) compared with radical nephrectomy. With the rapid development of surgical technology, minimally invasive PN (MIPN), encompassing robotic and laparoscopic approaches, has been widely applied in clinical practice, with proven advantages in reducing surgical complications and improving surgical precision (7).

Beyond its established role in angiography and tissue perfusion assessment within hepatic, breast, and other surgical specialties (8, 9), indocyanine green (ICG) fluorescence imaging has also evolved into a reliable intraoperative navigation tool in urological surgery (10, 11). In the context of PN, ICG facilitates the real-time visualization of tumor vascularity, collateral circulation, and ambiguous tumor margins, thereby optimizing surgical manipulation and enhancing procedural safety (10, 11). Accumulating evidence has substantiated the preliminary clinical benefits of ICG-assisted PN in routine practice.

In urological surgery, ICG can identify the arterial blood supply to renal tumors. When combined with selective vascular clamping, it mitigates ischemia in the normal renal parenchyma (12, 13). During resection for large or complex renal tumors, ICG assists surgeons in identifying collateral blood supply networks (11), thereby reducing estimated blood loss. Furthermore, when tumor margins are macroscopically indistinct, ICG can visualize these borders (11), potentially lowering the risk of positive surgical margins (14).

However, current evidence on ICG fluorescence guidance is largely restricted to low-complexity renal tumors, leaving its value across varying anatomical complexities (stratified by RENAL score) insufficiently defined. It remains unclear whether tumor complexity modifies the perioperative benefits of ICG, particularly regarding operative efficiency and renal function preservation in complex cases. To address this gap, this retrospective study evaluated perioperative outcomes between ICG and non-ICG cohorts undergoing MIPN, focusing on the differential impact of ICG across tumor complexity strata to guide its standardized clinical application.

## 2 Methods

### 2.1 Study design and participants

We retrospectively reviewed 77 consecutive patients with pathologically confirmed RCC who underwent MIPN at Peking University Shenzhen Hospital between April 2024 and August 2025. The cohort consisted overwhelmingly of robot-assisted partial nephrectomy (RAPN, *n* = 76, 98.7%), alongside one laparoscopic partial nephrectomy (LPN, *n* = 1, 1.3%). Patients were allocated to the ICG group (*n* = 31) or non-ICG group (*n* = 46) based on intraoperative ICG application (Figure 1). Allocation decisions depended on attending surgeons' clinical judgment based on tumor characteristics (size, location, depth, collateral supply), margin identification difficulty, equipment availability, and surgeon preference. All procedures were performed by three senior urologic oncologists, each with > 5 years of experience in minimally invasive PN and standardized ICG navigation training, adhering strictly to uniform institutional protocols.

**Figure 1:**
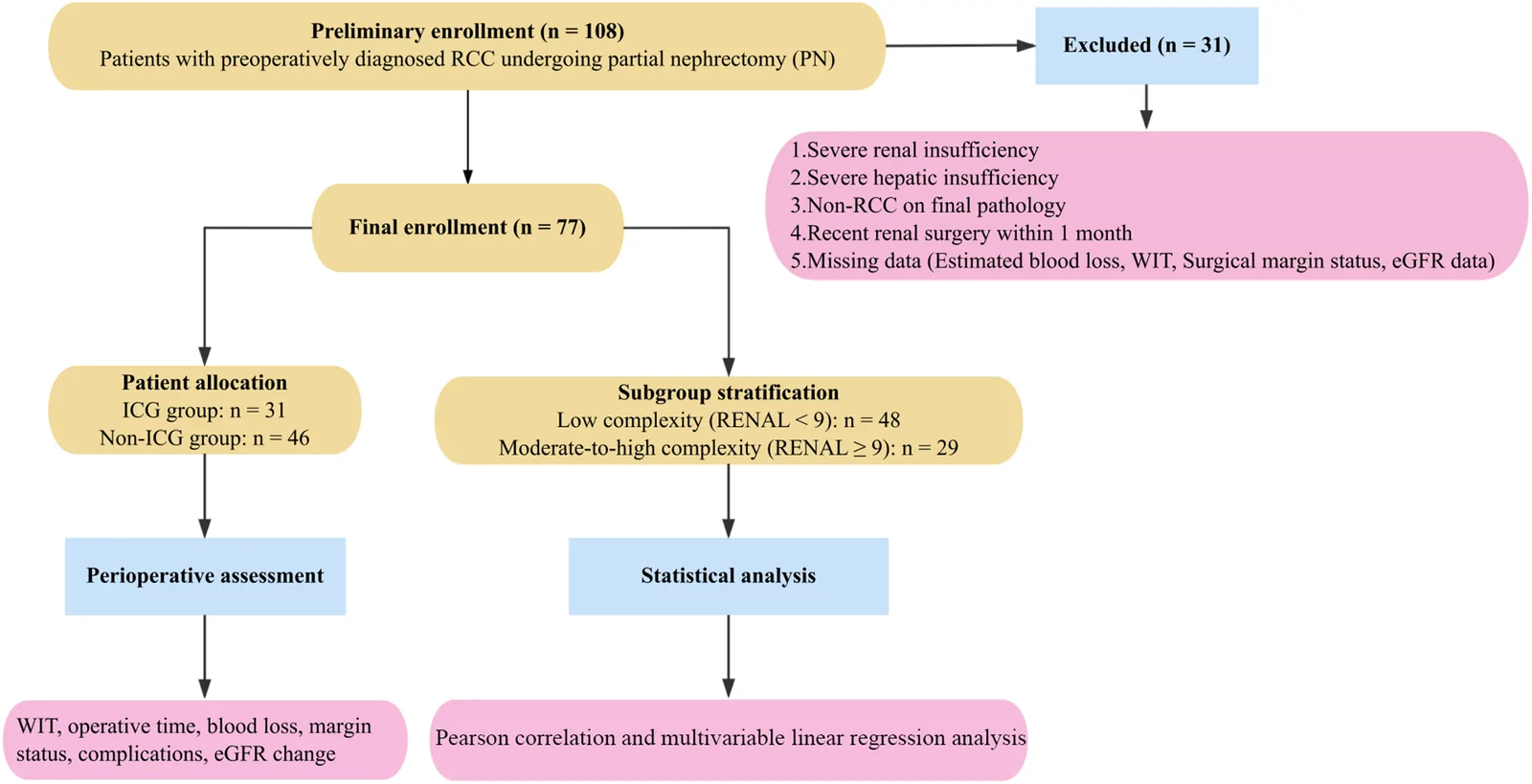
Patient recruitment and study flow diagram. A total of 108 consecutive patients with preoperatively suspected renal cell carcinoma (RCC) scheduled for partial nephrectomy were initially screened. Following the application of predefined exclusion criteria, 31 patients were excluded, resulting in a final analytical cohort of 77 patients. The cohort was divided into an indocyanine green (ICG) group (*n* = 31) and a non-ICG group (*n* = 46). Patients were further stratified by RENAL score into low-complexity (score < 9, *n* = 48) and moderate-to-high complexity (score ≥ 9, *n* = 29) subgroups. Collected perioperative parameters included warm ischemia time (WIT), operative time, estimated blood loss, surgical margin status, complications, and longitudinal changes in renal function. Pearson correlation and multivariable linear regression analyses were utilized to evaluate correlations and clinical differences across the RENAL score strata.

### 2.2 Inclusion and exclusion criteria

Inclusion criteria were as follows: one, pathologically confirmed RCC scheduled for PN; two, preoperative imaging assessment indicating tumor stage T1 or partial T2a; three, aged 18 years or older; and four, completion of a full set of preoperative routine examinations. Exclusion criteria included: one, a history of hypersensitivity to ICG; two, severe hepatic insufficiency (Child-Pugh class C); three, comorbid with severe acute conditions including active infection or coagulation disorders; or four, a history of other renal surgical procedures within the preceding one month.

### 2.3 Ethical approval and informed consent

Approved by the Institutional Review Board of Peking University Shenzhen Hospital (Approval number: 2026094), this retrospective study was granted a waiver of written informed consent due to its anonymized nature.

### 2.4 ICG usage and surgical procedure

The ICG dose was 0.25 to 0.5 mg/kg body weight, diluted in 10 mL of normal saline to achieve a final concentration of 2.5 mg/mL. A representative case demonstrating the complete intraoperative workflow is shown in Figure 2. Prior to ICG administration, the renal tumor and surrounding parenchyma were observed under standard white-light and near-infrared modes (Figures 2A,B). ICG was administered via peripheral intravenous injection after exposure of the renal tumor and prior to complete renal artery clamping. Immediately following injection, the near-infrared fluorescence imaging system was activated to observe renal arterial perfusion and facilitate selective arterial clamping (Figures 2C–F). Under fluorescence visualization, well-perfused renal parenchyma exhibited bright green fluorescence, whereas the renal tumor appeared as a hypovascular dark area with weak fluorescence signal, enabling real-time fluorescence-guided resection and precise surgical margin identification (Figures 2G–J). Following complete tumor enucleation, intense fluorescence in the final resection bed confirmed successful resection and adequate remaining parenchyma perfusion (Figures 2K,L).

**Figure 2:**
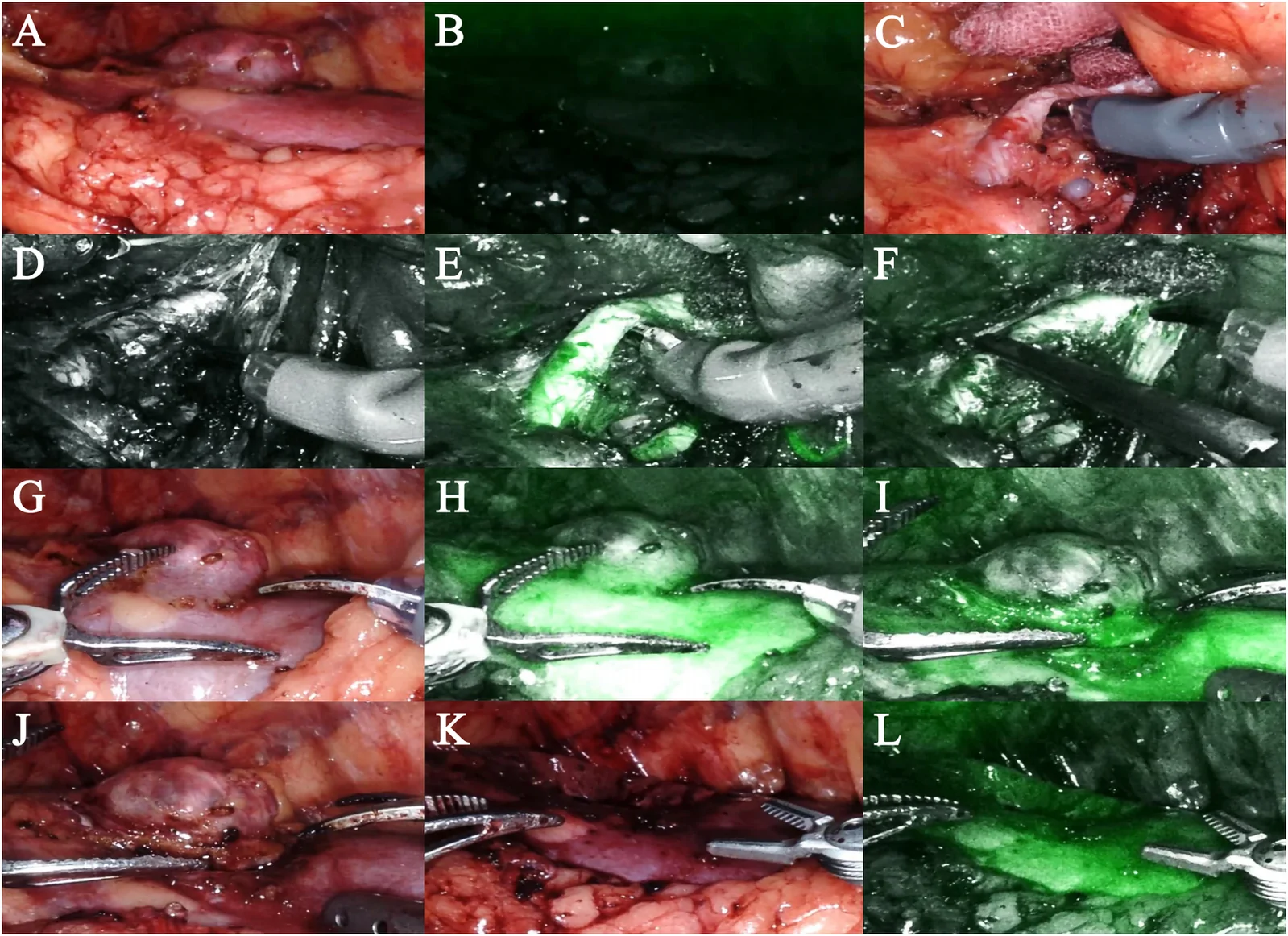
Sequential intraoperative ICG fluorescence-guided navigation during partial nephrectomy in a representative case. **(A,B)** Baseline visualization of a right-sided renal tumor prior to indocyanine green (ICG) administration under standard white-light mode **(A)** and near-infrared fluorescence mode **(B)**, showing comparable signals between the tumor and normal renal parenchyma with no distinct tumor demarcation. **(C–F)** Intraoperative manipulation and selective clamping of the right renal artery. Surgical view under white-light mode **(C)** and near-infrared mode **(D)** before ICG injection; near-infrared view after ICG injection **(E)** displaying bright green fluorescence in the right renal artery, indicating satisfactory arterial perfusion; and selective occlusion of the right renal artery **(F) (G–J)** Intraoperative surgical views after ICG administration. Tumor surgical field under white-light mode **(G)** and near-infrared mode **(H)** prior to resection, where well-perfused renal parenchyma exhibits bright green fluorescence and the hypovascular tumor appears as a dark area with weak signal; real-time ICG-fluorescence-guided resection enabling precise surgical margin identification **(I)**; and standard white-light view during resection **(J)**. **(K,L)** Final surgical resection bed following tumor enucleation under standard white-light mode **(K)** and corresponding near-infrared mode **(L)**, demonstrating intense fluorescence signal in the resection bed to confirm complete tumor excision and adequate perfusion of remaining renal parenchyma.

### 2.5 Clinical variables and data collection

Clinical data were retrospectively extracted from the hospital electronic medical record system. Demographic characteristics recorded included age, gender, and body mass index (BMI). We also assessed comorbidities, including hypertension, diabetes mellitus, and other chronic conditions. Tumor-related features included the maximum tumor diameter, TNM stage, RENAL score, Mayo Adhesive Probability (MAP) score, and tumor pathological subtype. Preoperative renal function parameters included serum creatinine, blood urea nitrogen, and estimated glomerular filtration rate (eGFR).

### 2.6 Observed outcome measures

Operative time was defined as the primary observational indicator to assess intraoperative efficiency related to ICG guidance. Secondary observational outcomes were grouped into three categories. First, serial renal function parameters, including serum creatinine, blood urea nitrogen, and eGFR measured at postoperative day 1, 1 week, 1 month, and 3 months; the preservation rate of eGFR on postoperative day 1 was defined as a key renal secondary metric. Second, other intraoperative surgical indexes, including warm ischemia time (WIT) and intraoperative blood loss. Third, postoperative recovery and complication indicators, including hospital length of stay, time to first flatus, and perioperative complications (intraoperative or postoperative bleeding, infection, urinary leakage) graded according to the Clavien-Dindo classification system.

### 2.7 Statistical methods

Statistical analyses were performed using IBM SPSS Statistics software (version 27.0.1). Continuous variables were described as mean ± standard deviation (SD) or median with interquartile range (IQR). Categorical variables were presented as counts and percentages. For unadjusted intergroup comparisons between the ICG group and non-ICG group, independent Student's *t*-test was adopted for normally distributed continuous indicators, while the Mann–Whitney U test was applied for skewed continuous variables. The *χ*^2^ test was used for categorical variables, and Fisher's exact test was substituted when cell counts were sparse in small subgroups. The primary endpoint of this study was operative time, reflecting intraoperative surgical efficiency under fluorescence guidance. Secondary endpoints included intraoperative metrics (warm ischemia time, estimated blood loss), postoperative surgical complications, and longitudinal renal functional parameters (eGFR preservation at postoperative day 1, 1 week, 1 month, and 3 months).

To investigate the independent impact of fluorescence guidance on operative efficiency, patients were stratified by anatomical complexity into low-complexity (RENAL score < 9) and moderate-to-high complexity (RENAL score ≥ 9) subgroups. The conventional RENAL nephrometry system originally defines three complexity tiers (low: 4–6, moderate: 7–9, high: 10–12); the dichotomized cutoff of ≥ 9 used herein represents a prespecified, self-defined stratification criterion. This cutoff was selected on clinical grounds that tumors with a RENAL score of 9 or above confer similarly elevated technical demands for tumor dissection, collecting system repair and renal parenchymal reconstruction, and dichotomization was adopted to maintain sufficient sample size and statistical power within each stratum given the moderate cohort size. Pearson correlation was used to assess the relationship between ICG use, operative time, and functional recovery.

For the primary continuous outcome of operative time, multivariable linear regression analysis was performed to identify the independent effect of intraoperative ICG use. The dependent variable was operative time, and the key independent variable was intraoperative ICG use (ICG group versus non-ICG group). Clinically validated confounders were adjusted in the full-cohort model, including tumor size, RENAL nephrometry score, and surgical approach (transperitoneal versus retroperitoneal). Covariate selection was based on established clinical evidence to avoid over-adjustment. In the exploratory subgroup analysis of high-complexity cases (RENAL score ≥ 9, *n* = 29), a parsimonious two-variable model (including intraoperative ICG use and a single prespecified covariate: tumor size for operative time and blood loss, or operative time for day-1 eGFR preservation rate) was fitted to prevent model overfitting associated with limited sample size. To ensure uniform model specifications and achieve valid between-stratum comparisons, the same two-variable model structure was applied to the low-complexity subgroup. A two-sided *P* value < 0.05 was considered statistically significant.

## 3 Results

### 3.1 Baseline clinical characteristics

To ensure a rigorous comparison between the two study cohorts, we first evaluated the baseline demographic and clinical characteristics to confirm inter-group balance. The study cohort comprised 77 patients, with 31 in the ICG group and 46 in the non-ICG group. Both groups were comparable regarding demographic data, including age (53.68 ± 14.52 vs. 53.89 ± 14.83 years), gender distribution (74% vs. 63% male), and body mass index (24.66 ± 7.40 vs. 24.73 ± 4.88 kg/m^2^). Clinical characteristics were well balanced, showing no significant differences in surgical approach (translumbar vs. transabdominal), surgical modality (robotic vs. laparoscopic), or preoperative laboratory indices. Preoperative imaging demonstrated no significant differences in tumor complexity, as indicated by the RENAL score (median 7 vs. 8, *P* = 0.131) and MAP score (median 1 vs. 1, *P* = 0.273). The mean maximum tumor diameter was 3.70 cm in the ICG group and 3.82 cm in the non-ICG group (*P* = 0.755). Pathological N and M stages were 0 for all participants (Table 1).

**Table 1:** Baseline demographic and clinical characteristics of the study cohort.

Variable	ICG group (*n* = 31)	Non–ICG group (*n* = 46)	*P* value
Age (years)	53.68 ± 14.52	53.89 ± 14.83	0.950
Male	23 (74)	29 (63)	0.437
BMI (kg/m^2^)	24.66 ± 7.40	24.73 ± 4.88	0.963
Hypertension	6 (19.4)	14 (30.4)	0.411
Diabetes	2 (6.5)	5 (10.9)	0.695
Surgeon, *n* (%)			0.172
Primary surgeon	29 (93.5)	37 (80.4)	
Other surgeons	2 (6.5)	9 (19.6)	
RENAL score	7 (7–8.5)	8 (7–9)	0.131
Low (4–6)	2 (6.5)	1 (2.2)	
Intermediate (7–9)	27 (87.1)	44 (95.7)	
High (10–12)	2 (6.5)	1 (2.2)	
MAP score	1 (0–1.0)	1 (0.25–2.0)	0.273
Low (0–1)	11 (35.5)	12 (26.1)	
Intermediate (2–3)	13 (41.9)	18 (39.1)	
High (4–5)	7 (22.6)	16 (34.8)	
Tumor diameter (CT, cm)	3.70 ± 1.45	3.82 ± 1.38	0.755
Preoperative Hb (g/L)	134.81 ± 16.50	136.63 ± 16.45	0.543
Preoperative Cr (*μ*mol/L)	77.00 ± 23.01	75.89 ± 20.15	0.625
Preoperative eGFR (mL/min/1.73 m^2^)	97.91 ± 24.17	92.84 ± 18.39	0.306
Tumor histology			0.270
ccRCC	27 (87.1)	35 (76.1)	
pRCC	1 (3.2)	5 (10.9)	
chRCC	0 (0)	2 (4.3)	
Other malignant	3 (9.7)	4 (8.7)	
Tumor stage			0.882
T1a	19 (61.3)	30 (65.2)	
T1b	11 (35.5)	14 (30.4)	
T2a	1 (3.2)	2 (4.3)	

Data are presented as *n* (%), mean ± SD or median (IQR) unless otherwise stated. BMI, body mass index; ccRCC, clear cell renal cell carcinoma; chRCC, chromophobe renal cell carcinoma; Cr, serum creatinine; CT, computed tomography; eGFR, estimated glomerular filtration rate; Hb, hemoglobin; ICG, indocyanine green; IQR, interquartile range; MAP score, Mayo adhesive probability score; pRCC, papillary renal cell carcinoma.

### 3.2 Perioperative surgical outcomes

To determine whether fluorescence guidance translates into improved procedural efficiency, we analyzed the primary and secondary perioperative outcomes. The primary finding was a significant reduction in operative time with ICG guidance. The mean operative time was 139.84 ± 36.84 min in the ICG group, which was significantly shorter than the 163.57 ± 44.80 min observed in the non-ICG group (*t* = −2.443, *P* = 0.017, 95% CI: −43.08 to −4.38). In the prespecified full-cohort multivariable linear regression model adjusted for tumor size, RENAL score, and surgical approach, intraoperative ICG use remained independently associated with a significant reduction in operative time (*B* = −23.808, 95% CI: −41.685 to −5.931, *P* = 0.010; Table 2). For other surgical parameters, the mean WIT was 24.03 ± 6.60 min in the ICG group compared to 25.30 ± 5.30 min in the non-ICG group (*P* = 0.353). The mean estimated blood loss was 34.52 ± 37.85 mL in the ICG group and 30.33 ± 30.23 mL in the non-ICG group (*P* = 0.592). Regarding oncological safety, the positive surgical margin rate was 0% in the ICG group and 4.35% (two cases) in the non-ICG group (*P* = 0.512). Furthermore, no significant differences were observed in maximum resection diameter or the minimum distance to the margin (Table 3).

**Table 2:** Multivariable linear regression analysis of factors associated with operative time in the overall cohort.

Variable	*B*	Standard error	95% CI	*P* value	VIF
Intraoperative ICG use	−23.808	8.968	−41.685 to −5.931	0.010	1.034
Tumor size	14.751	3.614	7.547 to 21.956	<0.001	1.358
RENAL score	−5.574	4.885	−15.312 to 4.164	0.258	1.322
Translumbar	−2.060	9.889	−21.774 to 17.655	0.836	1.096
Constant	153.614	37.781	78.298 to 228.930	<0.001	–
Overall model	–	–	–	<0.001	–

Dependent variable: operative time (min). *B*, unstandardized regression coefficient; 95% CI: 95% confidence interval; VIF, variance inflation factor; ICG, indocyanine green.

**Table 3:** Intraoperative and postoperative data of participants.

Variable	ICG group (*n* = 31)	Non–ICG group (*n* = 46)	*P* value
Surgical approach: RAPN	31 (100.0)	45 (97.7)	1.000
Translumbar	21 (67.7)	33 (71.7)	0.903
Operative time (min)	139.84 ± 36.84	163.57 ± 44.80	0.017
Estimated blood loss (mL)	34.52 ± 37.85	30.33 ± 30.23	0.592
WIT (min)	24.03 ± 6.60	25.30 ± 5.30	0.353
Resected max diameter (mm)	45.88 ± 18.46	48.42 ± 17.03	0.399
Hospital length of stay (days)	5.0 (4–8)	5.0 (4–10)	0.666
Time to first flatus (days)	1.0 (1–2)	1.0 (1–2)	0.957
Postoperative extubation time (days)	4.0 (3–5)	4.0 (3–5)	0.278
Positive surgical margins	0 (0)	2 (4.35)	0.512
Postoperative complications	5 (16.1)	9 (19.6)	0.771
Postoperative urine leak	0 (0)	0 (0)	
Postoperative bleeding	0 (0)	0 (0)	
Postoperative infection	5 (16.1)	9 (19.6)	
Clavien-Dindo	5 (16.1)	9 (19.6)	0.771
Clavien-Dindo I–II	5 (16.1)	9 (19.6)	
Clavien-Dindo III–IV	0 (0)	0 (0)	
Post-op Hb change at day one (g/L)	−9.32 ± 7.81	−13.1 ± 11.23	0.112
Preservation rate of post-op Hb at day one (%)	93.29 ± 5.32	90.51 ± 7.62	0.133
Post-op eGFR change at day one (mL/min/1.73 m^2^)	−5.41 ± 9.43	−5.64 ± 13.24	0.934
Preservation rate of post-op eGFR at day one (%)	94.58 ± 9.87	93.37 ± 14.69	0.689
Post-op Cr change at day one (μmol/L)	4.39 ± 12.78	7.28 ± 12.18	0.319
Post-op blood urea nitrogen change at day one (mmol/L)	−1.13 ± 0.99	−0.36 ± 1.19	0.004
Preservation rate of post-op eGFR at 1-week (%)	95.91 ± 9.01 (*n* = 6)	90.89 ± 14.44 (*n* = 17)	0.609
Preservation rate of post-op eGFR at 1-month (%)	94.94 ± 18.06 (*n* = 11)	86.84 ± 17.35 (*n* = 14)	0.396
Preservation rate of post-op eGFR at 3-months (%)	91.47 ± 10.81 (*n* = 12)	90.46 ± 16.51 (*n* = 23)	0.741

Data are presented as *n* (%), mean ± SD, or median (IQR) unless otherwise stated. Sample sizes for longitudinal eGFR at 1 week, 1 month, and 3 months reflect available cases at each follow-up window due to attrition. ICG, indocyanine green; RAPN, robot-assisted partial nephrectomy; IQR, interquartile range; WIT, warm ischemia time; Clavien-Dindo, Clavien-Dindo classification system; Hb, hemoglobin; Cr, serum creatinine; eGFR, estimated glomerular filtration rate.

### 3.3 Changes in renal function and functional recovery

To evaluate the functional safety and metabolic stability associated with this technique, we conducted a longitudinal assessment of renal function and recovery parameters. Postoperative renal function indices remained stable across both groups. On postoperative day one, the ICG group exhibited a mean serum creatinine increase of 4.39 ± 12.78 μmol/L, which did not significantly differ from the 7.28 ± 12.18 μmol/L increase in the non-ICG group (*P* = 0.319). The eGFR preservation rate was also similar between groups (94.58% ± 9.87% vs. 93.37% ± 14.69%; *P* = 0.689). A statistically significant difference was observed in the reduction of blood urea nitrogen (−1.13 ± 0.99 vs. −0.36 ± 1.19 mmol/L; *P* = 0.004), suggesting a smaller postoperative fluctuation in the ICG group. At 1 week, 1 month, and 3 months postoperatively, no significant inter-group differences in eGFR preservation rates and creatinine levels were observed among patients with available follow-up data, and values remained comparable to baseline in both groups (all *P* > 0.05). Due to substantial loss to follow-up at 1 week, 1 month, and 3 months (Table 3), findings regarding intermediate and long-term renal functional preservation are strictly exploratory and should be interpreted with caution.

### 3.4 Postoperative recovery and complications

To investigate the impact of indocyanine green on postoperative convalescence, we compared complication rates and recovery milestones between the two cohorts. Surgical safety was consistent between the two cohorts. No major complications, such as urinary leakage or bleeding requiring secondary intervention, were recorded. The incidence of Grade I to II complications according to the Clavien-Dindo classification was 16.1% (five cases) in the ICG group and 19.6% (nine cases) in the non-ICG group (*P* = 0.771). All complications involved minor infections that were successfully managed with conservative treatment. Recovery indicators, including mean postoperative ventilation duration, extubation time, and hospital length of stay (median five days in both groups), showed no significant differences (Table 3).

### 3.5 Stratified analysis and predictive models

To bridge the general perioperative findings with the underlying drivers of surgical efficiency, we performed stratified analyses based on the RENAL score (Table 4). In the low-complexity subgroup (RENAL score < 9, *n* = 48), ICG use significantly reduced the mean operative time (137.17 ± 37.41 vs. 164.56 ± 50.12 min; *P* = 0.038; Table 4). Pearson correlation analysis revealed a significant negative association between ICG use and procedural duration (*r* = −0.300, *P* = 0.039; Supplementary Table S1). In the multivariable regression model with uniform specification consistent with the high-complexity subgroup, ICG use showed a non-significant trend toward shorter operative time (*B* = −19.405, *P* = 0.099, 95% CI: −42.619 to 3.809; Supplementary Table S2), while larger tumor diameter was independently associated with prolonged operative duration (*B* = 19.130, *P* < 0.001, 95% CI: 8.917 to 29.342; Supplementary Table S2).

**Table 4:** Perioperative outcomes between ICG and non-ICG groups by RENAL score (<9 vs. ≥9).

Variable		ICG group (*n* = 31)	Non–ICG group (*n*=46)	*t*	*P* value
Operative time (min)	RENAL < 9	137.17 ± 7.41	164.56 ± 50.12	−2.130	0.038
	RENAL ≥ 9	147.50 ± 36.45	162.38 ± 38.72	−0.939	0.357
Estimated blood loss (mL)	RENAL < 9	21.74 ± 10.29	27.20 ± 18.82	−1.232	0.224
	RENAL ≥ 9	71.25 ± 61.05	34.05 ± 40.05	1.929	0.066
WIT (min)	RENAL < 9	22.57 ± 6.55	25.60 ± 5.56	−1.735	0.089
	RENAL ≥ 9	28.25 ± 4.95	24.95 ± 5.08	1.591	0.125
Post-op Hb change at day one (g/L)	RENAL < 9	−9.83 ± 8.39	−13.02 ± 12.65	1.023	0.311
	RENAL ≥ 9	−7.88 ± 6.08	−13.10 ± 9.58	1.426	0.168
Preservation rate of post-op Hb at day one (%)	RENAL < 9	93.17 ± 5.48	90.49 ± 8.39	1.298	0.201
	RENAL ≥ 9	93.66 ± 5.20	90.54 ± 6.79	1.170	0.254
Post-op eGFR change at day one (mL/min/1.73 m^2^)	RENAL < 9	−6.08 ± 9.66	−4.46 ± 10.56	−0.553	0.583
	RENAL ≥ 9	−3.48 ± 9.09	−7.04 ± 16.20	0.590	0.561
Preservation rate of post-op eGFR at day one (%)	RENAL < 9	94.21 ± 9.56	94.49 ± 11.54	−0.090	0.928
	RENAL ≥ 9	95.67 ± 11.35	92.05 ± 17.96	0.528	0.603

Data are presented as mean ± SD unless otherwise stated. WIT, warm ischemia time; Hb, hemoglobin; eGFR, estimated glomerular filtration rate. Comparisons between ICG and non-ICG groups were performed using independent Student's *t*-test.

In the moderate-to-high complexity subgroup (RENAL score ≥ 9, *n* = 29), although the initial comparison of means in Table 4 showed comparable operative times between groups (*P* = 0.357), Pearson correlation analysis revealed that operative time in this cohort was significantly negatively associated with the day one eGFR preservation rate (*r* = −0.421, *P* = 0.023; Supplementary Table S3). To isolate the specific impact of fluorescence guidance among these complex cases, we fitted a prespecified parsimonious two-variable exploratory multivariable regression model with identical specification to that used in the low-complexity subgroup. Within this model, larger tumor size independently predicted longer operative time (*B* = 12.100, *P* = 0.014, 95% CI: 2.306 to 21.894; Supplementary Table S4), while ICG use was not significantly associated with operative time (*B* = −28.960, *P* = 0.067, 95% CI: −60.086 to 2.166; Supplementary Table S4). Taken together, these exploratory stratified findings indicate that ICG use is associated with improved operative efficiency in low-complexity tumors, while its effect in high-complexity cases remains inconclusive after adjustment for tumor size. No formal interaction test was performed to confirm effect modification across complexity strata.

## 4 Discussion

In recent years, nephron sparing surgery has gained increasing clinical attention as the preferred treatment for localized RCC (5). The indications for this approach have expanded from strict clinical settings, such as a functional solitary kidney, to broader applications in localized tumors. This trend is closely associated with the continuous maturity and widespread adoption of MIPN, reflecting a shift toward prioritizing renal function preservation (15, 16). With the advancement of minimally invasive techniques, partial nephrectomy has emerged as the standard for tumors less than 4 cm and is selectively utilized for T2 stage lesions measuring 4 to 7 cm (5). The primary goal remains achieving complete resection while balancing oncologic cure and the preservation of healthy renal parenchyma to ensure long term functional stability (17, 18).

However, conventional intraoperative guidance often fails to meet the requirements of precision surgery, as visual inspection cannot determine the depth of parenchymal invasion. For deeply located tumors at the renal hilum, defining boundaries accurately is challenging, and manual palpation or ultrasound remains highly operator dependent. Improper palpation may lead to tumor rupture, while ultrasound deviations can cause misjudgment of tumor extent (19). In the present study, ICG fluorescence addressed these limitations by enabling real-time visualization of tumor margins and vascular anatomy, as demonstrated in Figure 2. This mechanism facilitated rapid tumor localization and renal pedicle management, thereby reducing repeated intraoperative exploration. Consequently, the operative time was significantly shorter in the ICG group (Tables 3, 4), whereas no significant differences were observed in postoperative recovery or complication rates (Table 3). This aligns with survey data from Narita's consensus meeting, which revealed that most urologists selectively reserve ICG for challenging renal tumors rather than routine practice. Such selective adoption is largely driven by concerns over additional reagent and equipment costs, coupled with the lack of definitive evidence demonstrating improvements in hard perioperative outcomes, including WIT, blood loss, and long-term eGFR (20).

To bridge the general findings with the underlying drivers of surgical efficiency, we performed stratified analyses based on the RENAL score. In the low-complexity subgroup (RENAL score < 9), ICG use significantly reduced the mean operative time (Table 4) and Pearson correlation analysis confirmed a significant negative association with procedural duration (*r* = −0.300, *P* = 0.039; Supplementary Table S1). In these cases, fluorescence guidance reduced exploration time by precisely identifying margins, which was a major contributor to efficiency. However, after adjustment for tumor size in the uniformly specified multivariable regression model, ICG use only showed a non-significant trend toward shorter operative time and was not an independent predictor of procedural duration (Supplementary Table S2). Consistently, multivariable regression further confirmed that ICG was not an independent predictor of the postoperative day-1 eGFR preservation rate or warm ischemia time in this stratum. Thus, fluorescence benefits in simple cases are primarily operational, as physiological compensations including parenchymal remodeling and contralateral renal upregulation likely stabilize short-term function (21).

In the moderate-to-high complexity subgroup (RENAL score ≥ 9), initial unadjusted comparisons showed no significant difference in operative time between groups (Table 4). However, in a prespecified parsimonious two-variable exploratory multivariable regression model adjusted with identical specifications to that applied in the low-complexity subgroup, larger tumor size was identified as an independent risk factor for prolonged operative time (*B* = 12.100, *P* = 0.014, 95% CI: 2.306 to 21.894; Supplementary Table S4), while the association between ICG use and operative time did not reach statistical significance (*B* = −28.960, *P* = 0.067; 95% CI: −60.086 to 2.166; Supplementary Table S4). Although adjustment for tumor size shifted the point estimate toward shorter operative time, the result remains inconclusive and statistically non-significant, likely constrained by the limited sample size (*n* = 29) and insufficient statistical power in this subgroup. Notably, due to factors such as complex tumor anatomy and increased difficulty in identifying blood supply (22, 23), ICG did not reduce intraoperative blood loss, which may also be attributed to the limited subgroup sample size. Given these limitations, all RENAL-stratified analyses should be interpreted as exploratory.

The discrepancy between the significant overall result and the non-significant subgroup findings is specific to the primary endpoint of operative time, and it is an expected statistical phenomenon rather than evidence of no clinical benefit. This pattern is primarily driven by the substantial reduction in statistical power after cohort stratification, especially in the moderate-to-high complexity subgroup with only 29 patients. Notably, the point estimate of operative time reduction in the high-complexity stratum was even larger than that in the overall cohort (*B* = −28.960 vs. −23.808 min), suggesting a potential clinical benefit that failed to reach statistical significance due to limited sample size.

Furthermore, our data established a critical link between procedural efficiency and functional preservation in complex clinical settings. In this subgroup, operative time was significantly negatively correlated with the day one eGFR preservation rate (*r* = −0.421, *P* = 0.023; Supplementary Table S3). Multivariable linear regression with uniform specification further quantified this independent association: each 1-min prolongation of operative time corresponded to a 0.2 percentage-point reduction in the eGFR preservation rate (*B* = −0.002, *P* = 0.029). Translated into a clinically relevant timeframe, this equates to an approximate 6 percentage-point decrease in eGFR preservation for every 30-min prolongation of operative time. Although shorter operative time correlated with higher early eGFR preservation in complex cases, whether ICG-mediated reduction in operative time directly or indirectly improves early postoperative renal function remains unknown and unproven in this cohort. In this study, the overall shortened operative time did not translate into broad differences in postoperative outcomes, likely because the reduction did not reach the critical threshold required to affect the systemic stress response (24). The statistically significant difference in blood urea nitrogen on postoperative day one likely represents a transient stress response rather than independent renal injury, as the values did not meet acute kidney injury criteria (25). The disappearance of these differences at one week and one month postoperatively further supports the transient nature of these metabolic changes.

This study has several limitations. First, as a single-center retrospective analysis, selection bias was inevitable, as ICG adoption gradually increased over time due to surgical learning curves and surgeon preference. Second, the modest total sample size (*n* = 77) and particularly small high-complexity subgroup (RENAL score ≥ 9) limited statistical power, constraining model stability and precluding propensity score matching or weighting methods. To mitigate this risk, parsimonious covariate sets with uniform specifications were adopted for all subgroup regression models. Although multivariable regression was applied to adjust for major confounders, residual confounding cannot be completely excluded. Third, substantial missing data in mid- and long-term follow-up introduced notable attrition bias. Renal function parameters at 1 week, 1 month, and 3 months postoperatively had high loss-to-follow-up rates, especially for the later time points, which may have compromised the accuracy of estimating the mid- to long-term renal functional impact of ICG guidance. Accordingly, all analyses of postoperative renal function beyond day 1 were treated as strictly exploratory to minimize the influence of attrition bias on core study inferences. Fourth, the cohort was heavily skewed toward robot-assisted surgery (98.7%), with only one laparoscopic case included, which precluded a meaningful subgroup comparison between robotic and laparoscopic approaches. Finally, the short 3-month follow-up is insufficient to evaluate long-term oncological and renal functional outcomes. Future prospective, multicenter studies with larger cohorts are needed to validate these findings.

## 5 Conclusion

The findings of this study suggest that the primary value of ICG in MIPN is optimizing intraoperative surgical efficiency and shortening operative time in the overall cohort. Whether ICG fluorescence guidance can effectively preserve or improve early postoperative renal function remains unknown. Thus, ICG should be defined primarily as a precision intraoperative auxiliary tool for efficiency rather than a proven functional intervention. This study confirms that tumor size and operative time are key factors affecting perioperative outcomes after MIPN. By facilitating precise lesion localization, ICG provides an evidence-based pathway for standardizing fluorescence-guided MIPN in urological practice.

## Data Availability

The raw data supporting the conclusions of this article will be made available by the authors, without undue reservation.
