# Supplementary material for: Evaluation of indocyanine green fluorescence guidance in minimally invasive partial nephrectomy stratified by tumor anatomical complexity: a single-center retrospective study
Source: Front Surg. 2026 Sep 18;13:1887270. doi: 10.3389/fsurg.2026.1887270 (PMC13630786; doi:10.3389/fsurg.2026.1887270)
Supplement: Supplementary file 1 [file Table1.docx]

| Table S1 Pearson correlation analysis (RENAL score < 9) | | | | | | | | | | | | |
| --- | --- | --- | --- | --- | --- | --- | --- | --- | --- | --- | --- | --- |
| Variable | 1 | 2 | 3 | 4 | 5 | 6 | 7 | 8 | 9 | 10 | 11 | 12 |
| 1. Intraoperative ICG use | 1.000 |  |  |  |  |  |  |  |  |  |  |  |
| 2. Age | 0.028 (0.852) | 1.000 |  |  |  |  |  |  |  |  |  |  |
| 3. Hypertension | -0.147 (0.317) | 0.538 (< 0.001) | 1.000 |  |  |  |  |  |  |  |  |  |
| 4. Diabetes | -0.054 (0.715) | 0.202 (0.168) | 0.193 (0.189) | 1.000 |  |  |  |  |  |  |  |  |
| 5. Translumbar | 0.022 (0.885) | 0.091 (0.541) | 0.133 (0.369) | 0.054 (0.714) | 1.000 |  |  |  |  |  |  |  |
| 6. Tumor size | -0.184 (0.212) | -0.110 (0.458) | -0.301 (0.038) | -0.178 (0.227) | -0.294 (0.042) | 1.000 |  |  |  |  |  |  |
| 7. Exophytic/endophytic properties | 0.080 (0.588) | -0.163 (0.269) | -0.215 (0.143) | -0.048 (0.744) | -0.005 (0.974) | -0.229 (0.117) | 1.000 |  |  |  |  |  |
| 8. Nearness | 0.158 (0.282) | 0.034 (0.817) | -0.119 (0.419) | -0.052 (0.727) | -0.376 (0.008) | 0.149 (0.312) | 0.077 (0.604) | 1.000 |  |  |  |  |
| 9. Anterior/posterior descriptor | 0.201 (0.170) | 0.187 (0.203) | 0.022 (0.882) | 0.036 (0.808) | 0.594 (< 0.001) | -0.036 (0.807) | 0.098 (0.509) | -0.250 (0.087) | 1.000 |  |  |  |
| 10. Location | 0.284 (0.051) | 0.100 (0.499) | 0.171 (0.246) | -0.115 (0.435) | 0.144 (0.329) | -0.110 (0.458) | -0.388 (0.006) | -0.003 (0.984) | 0.147 (0.318) | 1.000 |  |  |
| 11. Operative time | -0.300 (0.039) | -0.214 (0.144) | -0.187 (0.204) | -0.100 (0.497) | -0.078 (0.597) | 0.515 (< 0.001) | -0.079 (0.592) | 0.002 (0.989) | -0.195 (0.183) | -0.093 (0.531) | 1.000 |  |
| 12. WIT | -0.248 (0.089) | -0.069 (0.639) | -0.140 (0.344) | -0.220 (0.133) | -0.185 (0.209) | 0.317 (0.028) | 0.040 (0.789) | 0.169 (0.250) | -0.316 (0.029) | -0.018 (0.902) | 0.542 (< 0.001) | 1.000 |
| 13. Preservation rate of post-op eGFR at day one | -0.013 (0.928) | -0.187 (0.203) | -0.136 (0.355) | 0.110 (0.459) | 0.072 (0.626) | -0.376 (0.008) | 0.303 (0.037) | -0.139 (0.346) | -0.054 (0.715) | -0.013 (0.932) | -0.176 (0.231) | -0.141 (0.340) |
| Notes: Values outside parentheses are Pearson correlation coefficients; values in parentheses are P values; RENAL score, RENAL nephrometry score; ICG, indocyanine green; Nearness: the distance from the tumor to the collecting system; Location, the tumor’s position relative to the polar line; WIT, warm ischemia time. | | | | | | | | | | | | |
