# Supplementary material for: Evaluation of indocyanine green fluorescence guidance in minimally invasive partial nephrectomy stratified by tumor anatomical complexity: a single-center retrospective study
Source: Front Surg. 2026 Sep 18;13:1887270. doi: 10.3389/fsurg.2026.1887270 (PMC13630786; doi:10.3389/fsurg.2026.1887270)
Supplement: Supplementary file 2 [file Table2.docx]

| Table S2 Regression coefficients, model fit and significance test results of regression models for patients with RENAL score < 9 | | | |
| --- | --- | --- | --- |
| Variable | Model 1 | Model 2 | Model 3 |
| Overall model P value | 0.440 | 0.295 | < 0.001 |
| Intraoperative ICG use, t | -0.473 | -1.028 | -1.684 |
| B | -0.015 | -4.636 | -19.405 |
| 95% CI | -0.080 to 0.049 | -13.722 to 4.450 | -42.619 to 3.809 |
| P value | 0.638 | 0.310 | 0.099 |
| VIF | 1.099 | 1.035 | 1.035 |
| Operative time, t | -1.290 |  |  |
| B | -4.510 × 10⁻⁴ |  |  |
| 95% CI | -0.001 to 2.530 × 10⁻⁴ |  |  |
| P value | 0.204 |  |  |
| VIF | 1.099 |  |  |
| Tumor size, t |  | 0.996 | 3.773 |
| B |  | 1.977 | 19.130 |
| 95% CI |  | -2.020 to 5.974 | 8.917 to 29.342 |
| P value |  | 0.324 | < 0.001 |
| VIF |  | 1.035 | 1.035 |
| Notes: Model 1, preservation rate of post-op eGFR at day one; Model 2, estimated blood loss; Model 3, operative time; ICG, indocyanine green; B, unstandardized regression coefficient; 95% CI: 95% confidence interval; VIF, variance inflation factor; eGFR, estimated glomerular filtration rate; post-op, postoperative. | | | |
