# Supplementary material for: Evaluation of indocyanine green fluorescence guidance in minimally invasive partial nephrectomy stratified by tumor anatomical complexity: a single-center retrospective study
Source: Front Surg. 2026 Sep 18;13:1887270. doi: 10.3389/fsurg.2026.1887270 (PMC13630786; doi:10.3389/fsurg.2026.1887270)
Supplement: Supplementary file 3 [file Table3.docx]

| Variable | 1 | 2 | 3 | 4 | 5 | 6 | 7 | 8 | 9 | 10 | 11 | 12 |
| --- | --- | --- | --- | --- | --- | --- | --- | --- | --- | --- | --- | --- |
| 1. Intraoperative ICG use | 1.000 |  |  |  |  |  |  |  |  |  |  |  |
| 2. Age | -0.141 (0.465) | 1.000 |  |  |  |  |  |  |  |  |  |  |
| 3. Hypertension | -0.247 (0.197) | 0.371 (0.048) | 1.000 |  |  |  |  |  |  |  |  |  |
| 4. Diabetes | -0.168 (0.384) | 0.212 (0.269) | 0.286 (0.133) | 1.000 |  |  |  |  |  |  |  |  |
| 5. Translumbar | -0.201 (0.295) | 0.063 (0.747) | -0.131 (0.500) | 0.197 (0.305) | 1.000 |  |  |  |  |  |  |  |
| 6. Tumor size | 0.348 (0.064) | 0.101 (0.603) | -0.183 (0.342) | -0.124 (0.520) | -0.261 (0.171) | 1.000 |  |  |  |  |  |  |
| 7. Exophytic/endophytic properties | 0.081 (0.676) | -0.176 (0.361) | 0.052 (0.787) | 0.151 (0.435) | 0.089 (0.647) | -0.403 (0.030) | 1.000 |  |  |  |  |  |
| 8. Nearness | 0.117 (0.547) | 0.223 (0.244) | 0.076 (0.697) | 0.051 (0.791) | -0.137 (0.478) | 0.099 (0.609) | 0.055 (0.776) | 1.000 |  |  |  |  |
| 9. Anterior/posterior descriptor | 0.126 (0.514) | -0.257 (0.179) | -0.121 (0.530) | -0.083 (0.670) | -0.075 (0.701) | 0.149 (0.440) | 0.109 (0.574) | -0.071 (0.716) | 1.000 |  |  |  |
| 10. Location | -0.168 (0.384) | 0.283 (0.137) | -0.109 (0.574) | 0.463 (0.011) | 0.197 (0.305) | 0.103 (0.594) | -0.079 (0.682) | 0.051 (0.791) | -0.083 (0.670) | 1.000 |  |  |
| 11. Operative time | -0.178 (0.356) | 0.376 (0.045) | 0.072 (0.711) | -0.006 (0.977) | -0.198 (0.302) | 0.363 (0.053) | -0.181 (0.346) | 0.143 (0.460) | 0.095 (0.623) | -0.115 (0.553) | 1.000 |  |
| 12. WIT | 0.290 (0.128) | 0.220 (0.250) | -0.205 (0.285) | -0.260 (0.173) | 0.080 (0.680) | 0.252 (0.187) | 0.019 (0.921) | 0.255 (0.182) | 0.210 (0.274) | 0.088 (0.651) | 0.235 (0.220) | 1.000 |
| 13. Preservation rate of post-op eGFR at day one | 0.101 (0.602) | -0.104 (0.592) | -0.185 (0.336) | 0.060 (0.756) | -0.162 (0.401) | -0.185 (0.337) | 0.311 (0.100) | -0.012 (0.952) | -0.134 (0.489) | -0.031 (0.872) | -0.421 (0.023) | -0.142 (0.461) |

Table S3 Pearson correlation analysis (RENAL score ≥ 9)

Notes: Values outside parentheses are Pearson correlation coefficients; values in parentheses are P values; RENAL score, RENAL nephrometry score; ICG, indocyanine green; Nearness: the distance from the tumor to the collecting system; Location, the tumor’s position relative to the polar line; WIT, warm ischemia time.
