# Supplementary material for: Evaluation of indocyanine green fluorescence guidance in minimally invasive partial nephrectomy stratified by tumor anatomical complexity: a single-center retrospective study
Source: Front Surg. 2026 Sep 18;13:1887270. doi: 10.3389/fsurg.2026.1887270 (PMC13630786; doi:10.3389/fsurg.2026.1887270)
Supplement: Supplementary file 4 [file Table4.docx]

| Table S4 Regression coefficients, model fit and significance test results of regression models for patients with RENAL score ≥ 9 | | | |
| --- | --- | --- | --- |
| Variable | Model 1 | Model 2 | Model 3 |
| Overall model P value | 0.078 | 0.124 | 0.030 |
| Intraoperative ICG use, t | 0.150 | 1.486 | -1.913 |
| B | 0.010 | 30.665 | -28.960 |
| 95% CI | -0.123 to 0.143 | -11.754 to 73.084 | -60.086 to 2.166 |
| P value | 0.882 | 0.149 | 0.067 |
| VIF | 1.033 | 1.138 | 1.138 |
| Operative time, t | -2.304 |  |  |
| B | -0.002 |  |  |
| 95% CI | -0.003 to -1.920 × 10⁻⁴ |  |  |
| P value | 0.029 |  |  |
| VIF | 1.033 |  |  |
| Tumor size, t |  | 0.910 | 2.540 |
| B |  | 5.620 | 12.100 |
| 95% CI |  | -7.074 to 18.315 | 2.306 to 21.894 |
| P value |  | 0.371 | 0.014 |
| VIF |  | 1.138 | 1.138 |
| Notes: Model 1, preservation rate of post-op eGFR at day one; Model 2, estimated blood loss; Model 3, operative time; ICG, indocyanine green; B, unstandardized regression coefficient; 95% CI: 95% confidence interval; VIF, variance inflation factor; eGFR, estimated glomerular filtration rate; post-op, postoperative. | | | |
